# Biosynthesis and characterization of copper oxide nanoparticles from *Plumbago zeylanica* leaf extract for antibacterial and antioxidant activities

**DOI:** 10.1038/s41598-025-10700-z

**Published:** 2025-08-20

**Authors:** Hailemichael Tegenu Gebrie, Melesse Ababay Assege, Desta Shumuye Meshesha, Birhanu Ayalew Tebeje, Getaneh Worku Moges, Ayalew Temesgen Wodajo, Gizachew Mulugeta Manahelohe, Aderaw Anteneh Belew

**Affiliations:** 1 Department of Chemistry, College of Natural and Computational Sciences, University of Gondar, 196 Gondar, Ethiopia; 2 Department of Chemistry, College of Natural and Computational Sciences, Jigjiga University, P.O. Box 1020, Jigjiga, Ethiopia

**Keywords:** Antibacterial activity, Antioxidant activity, Copper oxide nanoparticles, Green synthesis, *Plumbago zeylanica* L.

## Abstract

In recent years, green synthesis has become a prevalent method for producing metallic oxide nanoparticles, preferred over traditional physical and chemical processes because of its low toxicity, cost-effectiveness, and environmental friendliness. Therefore, this study aimed to synthesize and characterize CuO NPs using *P. zeylanica* leaf extract, as well as to assess their total phytochemical content, in vitro antibacterial properties, and antioxidant activity. The synthesized CuO NPs were analyzed using UV–visible spectrophotometry, XRD, SEM, FTIR, and TGA/DTA. UV–visible spectroscopy revealed SPR peaks at 376 nm, confirming the formation of CuONPs, with a band gap energy of 3.22 eV indicating their semiconductor nature. FTIR analysis reveals the presence of Cu–O bonds around 528cm^−1^. XRD analysis further confirmed the monoclinic phase of CuO NPs, with an average crystallite size of 25.15 nm. The spherical shapes of the synthesized CuO NPs were determined using SEM analysis. TGA/DTA analysis revealed a weight loss of 19.3% within the temperature range of 21–600 °C. Along with this study, the antibacterial activity of the biosynthesized CuONPs was evaluated using an agar well diffusion assay against three gram-negative and two gram-positive. The results demonstrate that CuO NPs were more effective against gram-negative bacteria, showing inhibition zones of 19.33 mm, 20.30 mm, and 16.50 mm against *Escherichia coli*, *Pseudomonas aeruginosa,* and *Klebsiella pneumonia*, respectively. The antioxidant activity was evaluated, and IC_50_ values for DPPH assays of the synthesized CuO NPs, *P. zeylanica* leaf extract, and ascorbic acid were determined to be 123.77 ± 1.96, 97.28 ± 1.85, and 27.08 ± 0.15 μg/mL, respectively. These results indicate significant antioxidant and antibacterial activity for CuO NPs.

## Introduction

Nanotechnology has recently emerged as a multidisciplinary field of scientific and technological research, focusing on developing new techniques for creating nanoparticles (NPs) with the appropriate sizes, usually up to 100 nm in diameter, and specific shapes^1^. Unlike their bulk counterparts, nanoscale materials exhibit unique size and surface-to-volume ratios, which significantly enhance their mechanical properties, melting points, electrical and thermal conductivity, and catalytic activity^2,3^.

Nowadays, metal oxide NPs are widely utilized across various fields, including medicine, photocatalysis, photovoltaics, industry, agriculture, drug-gene delivery, batteries, biosensors, construction, food packaging, automobiles, and environmental remediation, due to their unique properties^4^. These metal oxide NPs, including Fe_2_O_3_, Fe_3_O_4_, MgO, TiO_2_, ZnO, and CuO NPs, exhibit significant biological activity even at very low concentrations due to their unique physicochemical properties^5^.

Among them, CuO NPs have gained significant attention from researchers for solar cells, biodiesel production, water remediation, supercapacitors, catalysis, and antibacterial and anticancer therapies^6^. CuO NPs are p-type semiconductors characterized by a narrow band gap, making them highly effective for applications in heterogeneous catalysis, textiles, batteries, nanomedicine, energy storage, drug delivery, gas sensors, and wood preservation^4,5^. They also have antifungal, anti-inflammatory, anti-diabetic, antioxidant, and antifouling activities^4^. This is because they have excellent thermal stability, biocompatibility, diverse nanoscale morphologies, ease of synthesis, large surface area, high conductivity, enhanced oxygen adsorption capacity, abundant availability, and cost-effectiveness compared to other noble metals^7^.

CuO NPs have been synthesized using various methods, including lithography^8^, laser ablation^9^, chemical precipitation methods^10^, electrochemical methods^11^, hydrothermal methods^12^, and chemical bath deposition^13^, as well as biological approaches. However, most conventional methods for CuO NP synthesis are complex, costly, time-consuming, and involve toxic chemicals, limiting their practical use^14^. For this reason, green synthesis methods have been developed to use safe reagents, reduce energy consumption, and produce non-toxic and environmentally friendly products and byproducts^15^. Recently, various biological sources, including algae, bacteria, plants, fungi, and yeast, have been used for the synthesis of CuO NPs^3^. Among these, plant extracts are preferred due to their stability, availability, and richness in metabolites, which make the synthesis faster and simpler compared to methods involving bacteria or fungi^16^. Moreover, numerous scientific studies have reported the green synthesis of CuO NPs using a variety of plant extracts, including *Caesalpinia bonducella*^17^*, Aerva javanica*^18^, *Bacopa monnieri*^5^, *Eupatorium adenophorum*^19^*, Elsholtzia blanda*^4^, *Morinda citrifolia*^3^, *Abelmoschus esculentus*^20^*, Acer palmatum*^15^, *Tribulus terrestris*^21^*, Ephedra alata*^7^*,* and *Jasminium sambac*^22^.

Although CuO NPs have been successfully synthesized using a variety of plant extracts, the potential application of *Plumbago zeylanica* L. *(P. zeylanica*) in this context remains largely unexplored. In the present study, CuO NPs were biosynthesized using the leaf extract of *P. zeylanica*, a plant commonly known as doctorbush or Ceylon leadwort. This plant belongs to the family *Plumbaginaceae* and is widely distributed in tropical and subtropical regions, including Asia, Australia, and Africa^23^*. P. zeylanica* is a traditional medicinal plant used to treat chronic coughs, neural disorders, colds, intestinal worms, diarrhea, skin diseases, gonorrhea, syphilis, tuberculosis, edema, and injuries^24–26^. Various parts of the plant have been demonstrated to have therapeutic properties, including antioxidant^27^, anticancer^24^, antimicrobial^25^, and nephroprotective activities^28^. *P. zeylanica* contains diverse chemical compounds, including naphthoquinones, alkaloids, saponins, flavonoids, tannins, glycosides, coumarins, steroids, phenolic compounds, triterpenoids, carbohydrates, fixed oils, fats, and proteins^29^. These components could serve as stabilizing, reducing, and capping agents in the transformation of metal salts to CuO NPs. Due to the diverse applications, NPs synthesis was conducted using the leaf *P. zeylanica* extract. Copper was selected for this study because of its remarkable biological properties and excellent electrical conductivity.

A literature review highlights limited research on the use of *P. zeylanica* phytochemicals for the biosynthesis of CuO NPs, with no reports specifically investigating its leaf extract in Ethiopia for antioxidant and antibacterial activities. In this study, novel CuO NPs were synthesized using *P. zeylanica* leaf extract by optimizing various reaction parameters, and their antibacterial and antioxidant properties were evaluated. The synthesized CuO NPs were characterized via UV–Vis, XRD, FT-IR, SEM, and TGA and evaluated for their in vitro antibacterial and antioxidant activities. However, the study does not include further in vivo validation or mechanistic studies.

## Materials and methods

### Chemicals and reagents

In this study, the following chemicals and reagents were used: gallic acid (Sisco Maharashtra, India), L-ascorbic acid (99%, Loba Chemie Pvt. Ltd., Mumbai, India), 2,2-diphenyl-1-picrylhydrazyl (DPPH, CHD Ltd., New Delhi, India), Folin–Ciocâlteu reagent (Sigma-Aldrich, Johannesburg, South Africa), quercetin (Loba Chemie, India), CuSO_4_⋅5H_2_O (GTECH, China), NaOH (99%, Loba Chemie, India), HCl (37%, Pentokey, India), H_2_SO_4_ (98%, Blulux, India), AlCl_3_ (Loba Chemie, India), sodium carbonate, Muller–Hinton agar (Siscico Pvt. Ltd., India), potassium ferric cyanide (BHD Limited Poole England), ciprofloxacin, and potassium acetate (99%, Loba Chemie, India). All chemical reagents used were analytical grade; ultrapure deionized water was employed throughout the experiment for the green synthesis of CuONPs.

### Collection and preparation of plant extract

Fresh *P. zeylanica* leaves were collected in April 2024 from Tis Abay, West Gojjam, Ethiopia. Geographically, Tis Abay is located at a latitude of 11° 29′ 26.92″  N and a longitude of 37° 35′ 15.74″  E, at an elevation of 1645 m above sea level. The leaves were authenticated by Mr. Abiyu Enyew, a botanist at the University of Gondar, and a voucher specimen, MAA001/2024 was deposited in the University of Gondar Herbarium. The leaves were thoroughly cleaned with tap water, followed by distilled water, to remove dust and other impurities. They were then air-dried in a shaded area at room temperature for 14 days to eliminate moisture content. After drying, the leaves were finely ground using a mechanical grinder and stored in an amber bottle.

The aqueous extraction of *P. zeylanica* leaves was carried out following a protocol adapted from^30^ with slight modifications. Twenty grams of powdered leaves were mixed with 1000 mL of ultrapure deionized water in a 2000 mL flask. The flask was covered with aluminum foil to protect it from light, shaken for 90 min, and heated at 60 °C for 1 h on a magnetic stirrer to facilitate the extraction of phytochemicals from the plants. The resulting mixture was then filtered using Whatman No. 1 filter paper, centrifuged at 350 rpm for 10 min, and the filtrate was stored at 4 °C for use in CuO NPs synthesis.

### Qualitative phytochemical analysis

The qualitative phytochemical analysis of the aqueous extract of *P. zeylanica* leaves was carried out to identify the various secondary metabolites, such as alkaloids, cardiac glycosides, saponins, flavonoids, phenols, tannins, terpenoids, anthraquinones, steroids, and coumarins^31,32^.

### Biosynthesis of CuO NPs by using *Plumbago zeylanica* extract

**Fig. 1:**
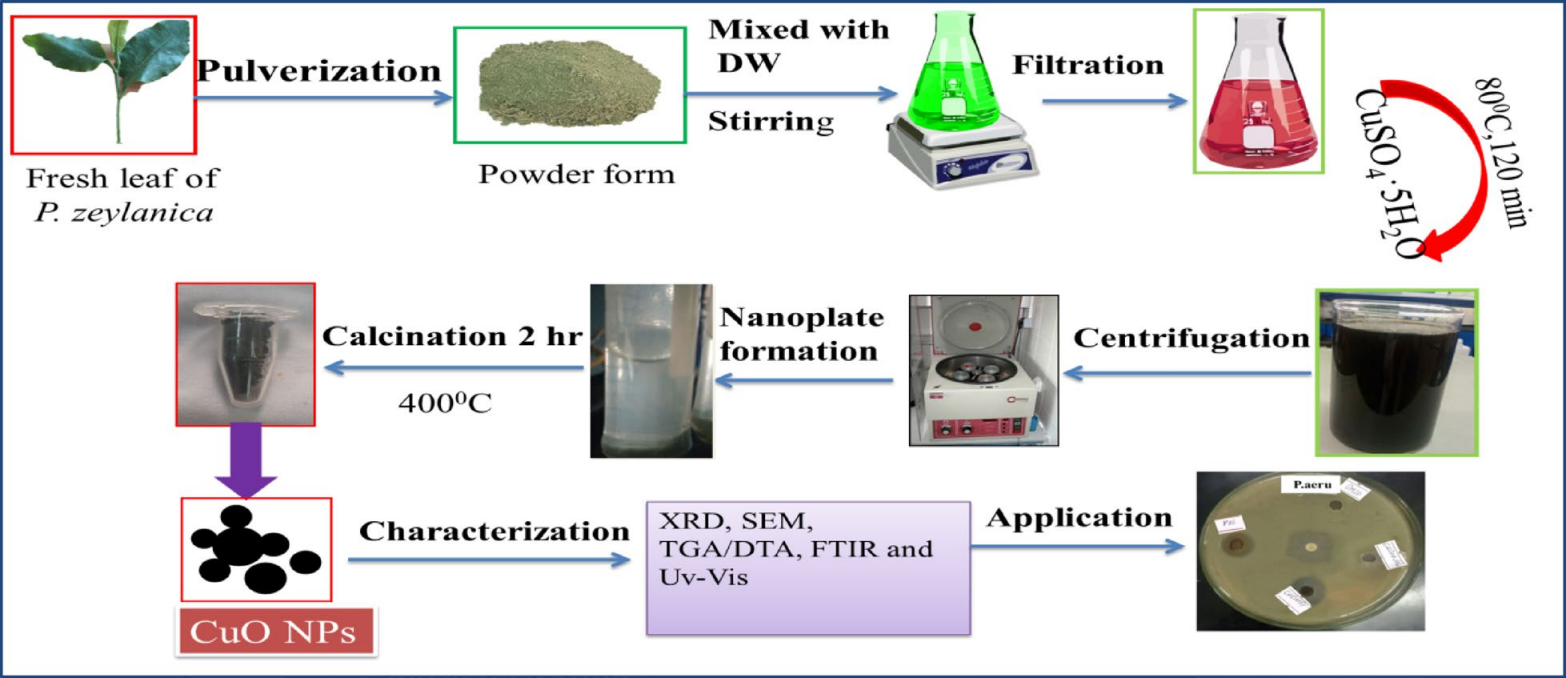
A schematic flowchart for the preparation of *P. zeylanica* leaf extract.

CuONPs were synthesized using *P. zeylanica* leaf extract as a capping agent and CuSO_4_·5H_2_O as a precursor, following a modified method^33^. The synthesis parameters, including the concentration of CuSO_4_·5H_2_O, the volume ratio of *P. zeylanica* leaf extract, pH, temperature, and incubation time, were optimized. The resulting black CuO NPs solution was centrifuged at 4000 rpm for 15 min, washed with ultrapure deionized water and ethanol, then dried at 70 °C, calcined at 400 °C, and stored in appropriate containers, as shown in Fig. 1

### Characterization of copper oxide nanoparticles

Black CuO NPs were obtained after calcination at 400 °C for 2 h. They were analyzed using UV–Vis spectrophotometry (Abron, India), FTIR spectroscopy (Perkin Elmer, 65, USA), SEM, and TGA/DTA (DTG-60H, Shimadzu Co., Japan) techniques. The average crystal size of the biosynthesized CuONPs was determined through XRD analysis. The XRD measurements were conducted using a Shimadzu XRD-7000 (Shimadzu Co., Japan), equipped with a Cu target to generate CuKα radiation (λ = 0.154060 nm). Diffraction data were collected over a 2θ range of 10–80°. The measurements were conducted at room temperature, with an accelerating voltage of 40 kV and an applied current of 30 mA.

### Quantitative phytochemical analysis of CuO NPs and leaf extract

#### Determination of total phenolic content

Total phenolic content (TPC) was determined using the Folin-Ciocalteu method^34^, with minor modifications. A 0.5 mL sample was combined with 3 mL of ultrapure deionized water and 0.5 mL of Folin-Ciocalteu reagent and then incubated for 5 min. After incubation, 1.5 mL of 7.5% Na_2_CO_3_ was added to the mixture, which was then incubated in the dark for 90 min. The absorbance of the sample was measured at 765 nm using a UV–Vis spectrophotometer. The TPC was calculated by referencing a gallic acid calibration curve (2.5–250 µg/mL) and expressed as mg/g dry weight. Experiments were performed in triplicate.

#### Determination of total flavonoid content

Total flavonoid content (TFC) was measured using a colorimetric assay^35^ with minor adjustments. Briefly, 0.5 mL sample or standard solution was combined with 1.5 mL of ultrapure deionized water, 0.1 mL of 10% AlCl_3_, 0.1 mL of 1 M CH_3_CO_2_K, and 2.8 mL of ultrapure deionized water. The mixture was then incubated at room temperature for 30 min, and the absorbance was measured at 415 nm using a UV–Vis spectrophotometer. TFC was calculated using a quercetin calibration curve (10–400 μg/mL). The experiments were conducted in triplicate.

### Antioxidant assays

The *P. zeylanica* leaf extracts and the synthesized CuO NPs were evaluated for their in vitro antioxidant activity using two commonly employed radical scavenging assays: DPPH and FRAP assays.

#### DPPH radical scavenging assays

The antioxidant capacity of the leaf extract and green-synthesized CuO NPs was measured using the DPPH method, as outlined by^36^ with minor modifications. A 0.1 mM DPPH solution was prepared in methanol and stored for 3 h for stability. The absorbance was adjusted to 1.1 ± 0.4 using a spectrophotometer. Stock solutions of CuO NPs, *P. zeylanica* leaf extract, and ascorbic acid were prepared by dissolving 25 mg of each in 25 mL of an appropriate solvent to obtain the required concentrations for the assay. In test tubes, 0.5 mL of each sample (ranging from 10 to 1000 μg/mL) was mixed with 2.5 mL of DPPH solution. The control sample contained methanol instead of the test samples. The samples were incubated for 30 min, and the absorbance was measured at 517 nm using a UV–Vis spectrophotometer. The free radical scavenging activity was determined using the formula provided in Eq. (1).

1 $${\text{\% Inhibiton }} = \frac{{\text{Ac - As}}}{{{\text{Ac}}}}{*100}$$

where A_c_ absorbance of the control, and A_s_ absorbance of the sample.

After calculating the % inhibition of the *P. zeylanica* leaf extract and CuO NPs, the IC_50_ values for their DPPH free radical scavenging activities were determined using the GraphPad Prism statistical model.

#### Ferric reducing antioxidant power (FRAP) assay

With minor adjustments to the method, the ferric-reducing power of the leaf extracts and synthesized CuO NPs was determined^37^. In brief, 1.0 mL of sample (100–1000 μg/mL) was combined with 2.5 mL of 0.2 M phosphate buffer (pH 6.6) and 2.5 mL of 1% potassium ferricyanide, followed by incubation at 50 °C for 20 min. After the addition of 2.5 mL of 10% trichloroacetic acid, the mixture was centrifuged at 5000 rpm for 10 min. The resulting upper layer (2.0 mL) was mixed with 2.0 mL of deionized water and 0.4 mL of 0.1% ferric chloride, and absorbance was measured at 700 nm after 10 min. Ascorbic acid was used as the standard.

### Antibacterial activity

The antibacterial activity of *P. zeylanica* leaf extract and CuO NPs was evaluated against S*treptococcus pneumoniae*, *Staphylococcus aureus*, *Pseudomonas aeruginosa*, *Escherichia coli*, and *Klebsiella pneumoniae* using the agar well diffusion method^33^. Bacterial cultures were grown in nutrient broth at 37 °C for 24 h. Sterilized Mueller Hinton agar plates were inoculated with bacterial samples, and wells were made using a 6-mm cork borer. Samples (150 mg) were dissolved in 3 mL DMSO, and 100 µL was added to each well. Ciprofloxacin (5 µg/disk) and DMSO served as positive and negative controls, respectively. Plates were incubated at 37 °C for 24 h, and the zone of inhibition was measured. All experiments were performed in triplicate.

### Statistical data analysis

All experiments were conducted in triplicate, and the results were presented as mean ± standard deviation. Statistical analysis was carried out using Microsoft Excel 2019, while graph analysis was conducted using Origin Pro (version 21.1) and ImageJ software. The IC_50_ was calculated using GraphPad Prism (version 9.3.1.47). A p-value of less than 0.05 was considered statistically significant.

## Results and discussions

### Phytochemical screening of plant extracts

**Fig. 2:**
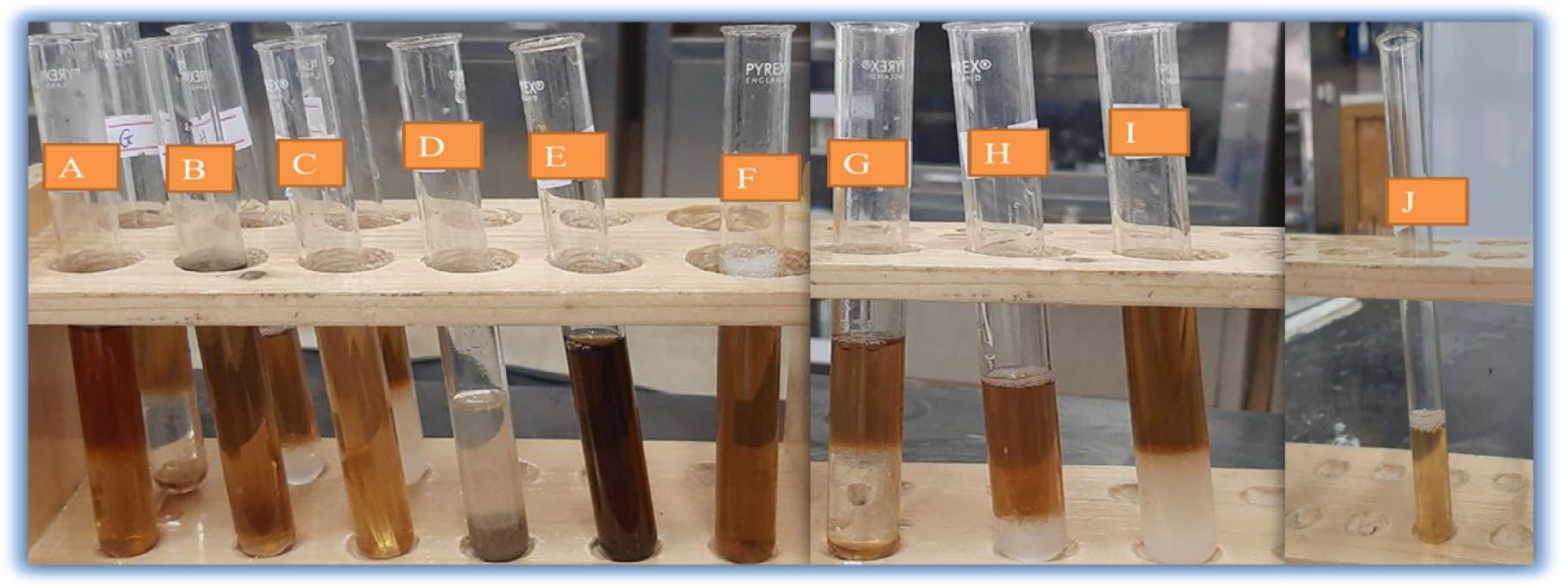
Aqueous extract of *P. zeylanica* extract phytochemical analysis (**a**) Alkaloid, (**b**) Cardiac glycosides, (**c**) Flavonoid, (**d**) Tannin, (**e**) Phenol, (**f**) Saponins, (**g**) Steroids, (**h**) Terpenoids, (**i**) Anthraquinones, and (**j**) Coumarins.

In this study, phytochemical tests of *P. zeylanica* leaf aqueous extract showed positive results for tannins, phenols, saponins, flavonoids, terpenoids, steroids, coumarins, and cardiac glycosides. These compounds are likely involved in reducing and stabilizing CuO NPs during synthesis. The presence of these phytochemicals, which include alkaloids, flavonoids, phenol, and others, plays a key role in the synthesis of CuO NPs^38^. The results of the presence or absence of phytochemicals in the aqueous extract of *P. zeylanica* leaves are presented in Table 1 and Fig. 2.

**Table 1:** Preliminary phytochemical constituent in aqueous extracts of *P. zeylanica* leaves.

S/N	Phytoconstituents	Reagent/test	Observation
1	Alkaloid	Wagner	−
2	Cardiac glycosides	Keller-Kiliani	+
3	Flavonoid	Shinoda test	+
4	Tannin	Lead acetate	+
5	Phenol	Ferric chloride	+
6	Saponins	Foam test	+
7	Steroids	Liebermann-Burchard	+
8	Terpenoids	Salkowski test	+
9	Anthraquinones	Bontrager test	−
10	Coumarins	10% NaOH	+

Key − absent + present.

### Visual observation

**Fig. 3:**
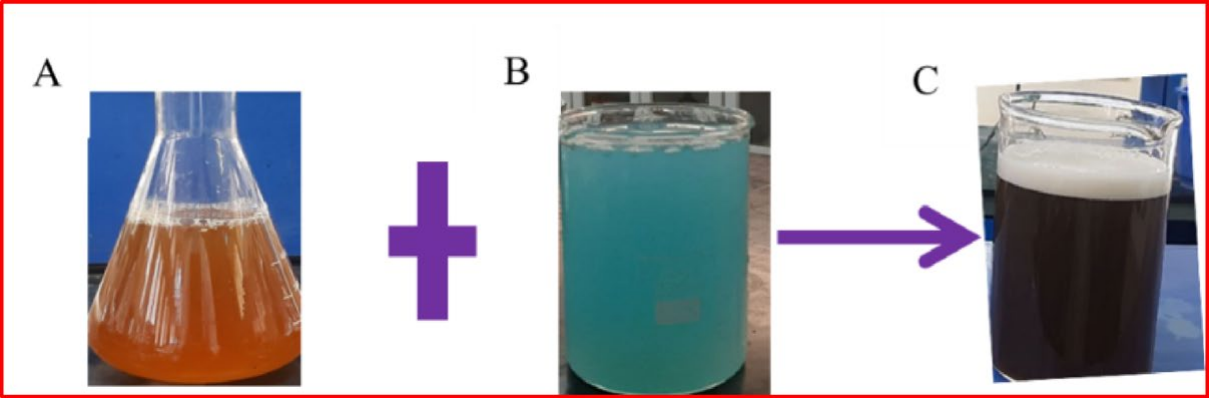
Color of (**a**) *P. zeylanica* (**b**) CuSO_4_·5H_2_O solution (**c**) precursor solutions with aqueous extract of *P. zeylanica* leaf.

The present study illustrated that the formation of CuO NPs using *P. zeylanica* extract was an eco-friendly, less expensive and nontoxic method. The visual examination confirmed the preliminary formation of CuO NPs. The formation of CuO NPs was easily recognized by the color change of the solution. As shown in Fig. 3, when *P. zeylanica* was added to the CuSO_4_·5H_2_O, the mixing exhibited a color change from blue (aqueous CuSO_4_·5H_2_O) and pink (pure extract color) to reddish-brown during the reaction. This color change indicates the biosynthesis of CuONPs, which is caused by the excitation of surface plasmon resonance in metal nanoparticles, signifying the formation of CuONPs.

### Optimization of the reaction parameters for the synthesis of CuONPs

The experimental parameters, including the concentration of CuSO_4_·5H_2_O, incubation time, temperature, volume of extract, and pH, were adjusted for optimal CuO NP formation.

#### Effect of CuSO_4_·5H_2_O concentration

**Fig. 4:**
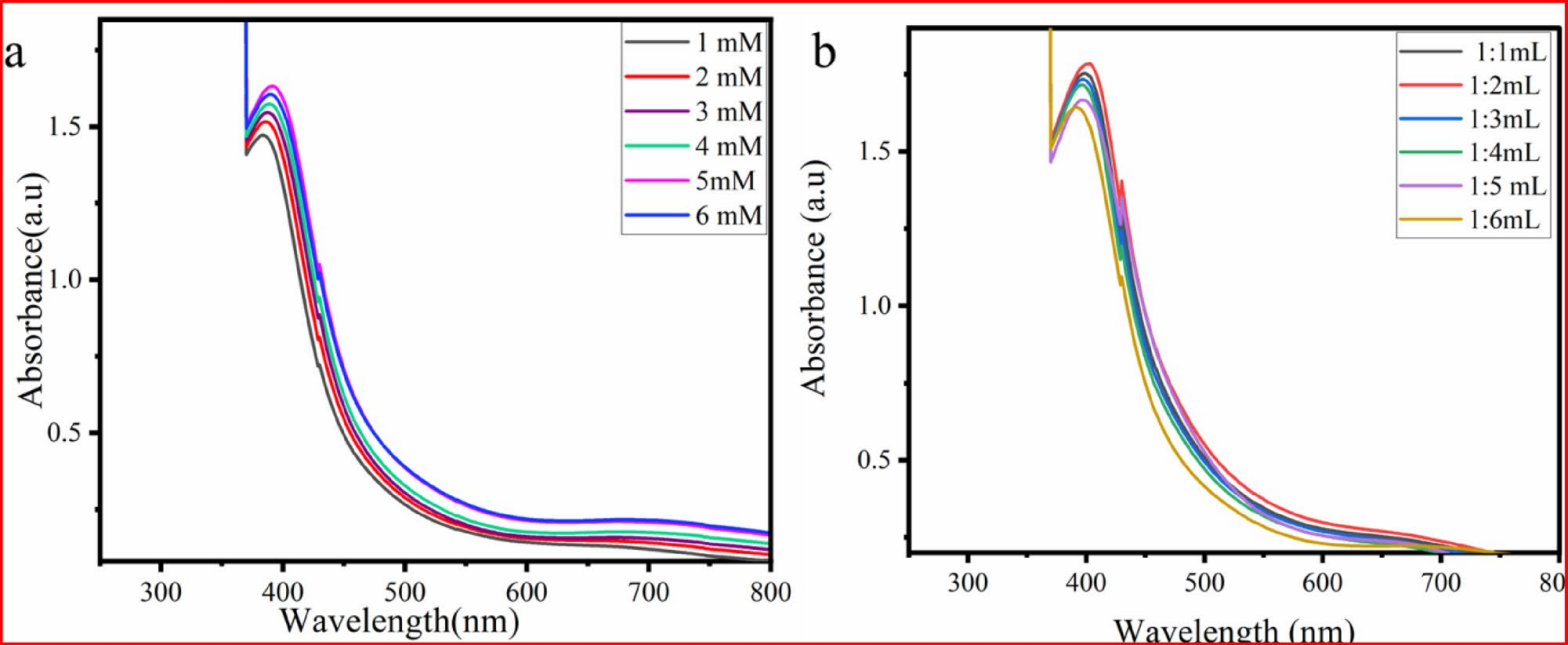
UV–visible spectra of CuONPs with different (**a**) CuSO_4_·5H_2_O concentrations, and (**b**) volumes *P. zelenica* of extract.

The effect of CuSO_4_·5H_2_O concentration on CuO NP synthesis was monitored using a UV‒vis spectrophotometer. In this study, the CuSO_4_·5H_2_O concentrations ranging from 1 to 6 mM were tested. The absorption spectrum revealed that increasing the concentration of CuSO_4_·5H_2_O up to 5 mM led to stronger absorption intensity. However, at 6 mM (Fig. 4a**)**, a decrease in the absorption spectrum indicated nanoparticle aggregation^39^. Therefore, the optimal concentration was found to be 5 mM, showing stronger absorption. This study suggests that higher metal ion concentrations beyond a certain point lead to reduced nanoparticle production due to faster agglomeration^40^.

#### Effect of extract volume

The synthesis of CuONPs using plant extracts is highly influenced by the types of biomolecules present in the extracts and the volume used. The amount of plant extracts in NP synthesis plays a crucial role in determining the duration of the process^41^.

In this study, the volume ratio of *P. zeylanica* leaf extract to the precursor solution was tested from 1:1 mL to 1:6 mL. As shown in Fig. 4b, absorbance increased as the volume ratio of *P. zelenica* leaf extract to the precursor solution increased from 1:1 mL to 1:2 mL. However, beyond the 1:2 mL ratio, the absorption intensity decreased. This suggests that exceeding a certain threshold in the extract volume may lead to NP aggregation, which reduces absorption intensity^42^. These results revealed that the optimum volume ratio for the synthesis of CuONPs was 1:2 mL, as confirmed by a UV–visible absorption analysis. The increase in absorbance at this ratio can be attributed to the presence of more phytochemicals and bioactive compounds, which bind to the surface of CuO NPs^40^.

#### Effect of temperature

**Fig. 5:**
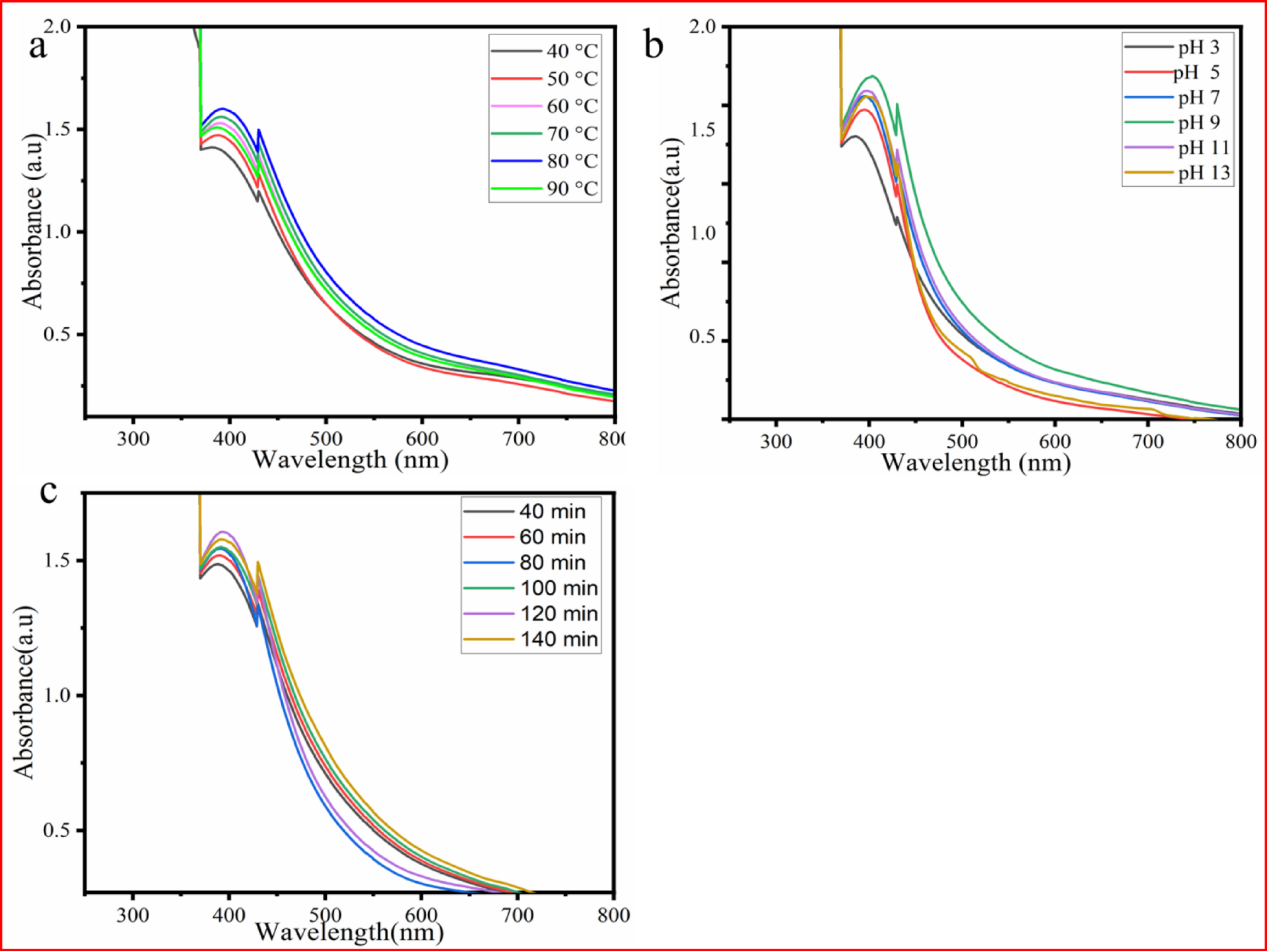
UV–visible spectra of CuONPs prepared with different (**a**) temperature, (**b**) pH, and (**c**) incubation times.

Temperature plays a crucial role in the synthesis of CuO NPs. The effect of temperature was optimized within the range of 40–90 °C. As shown in Fig. 5a, the absorbance of the solution increases with increasing temperature from 40 to 80 °C, indicating a higher abundance of CuONPs. However, beyond 80 °C, the absorption begins to decrease, suggesting a reduction in the yield of CuONPs. These findings confirm that 80 °C was the optimal temperature for maximum CuO NP yield.

Additionally, as the temperature reaches 90 °C, the interaction between secondary metabolites and the synthesis process decreases, destabilizing the phytochemicals and slowing CuO NP formation. This leads to unstable agglomeration of the nanostructures and a decrease in absorbance^38,40^.

#### Effect of pH

The pH significantly affects the morphology and size of CuO NPs, with adjustments allowing for straightforward control over the size of the resulting product^15^. In this study, the pH ranged from 3 to 13. As shown in Fig. 5b, increasing the pH from 3 to 9 increased absorption intensity, indicating enhanced nanoparticle formation. However, further increasing the pH to 13 led to a decrease in absorption intensity, suggesting reduced CuO NP formation. Therefore, pH 9 was chosen as the optimal value, as it exhibited the highest absorption intensity in UV–visible spectra. A higher pH promotes more nucleation centers, leading to increased CuONP formation^43^.

#### Effect of incubation time

Incubation time plays a crucial role in determining the quality, morphology, and yield of CuONPs. In the present study, increasing the incubation time from 40 to 120 min increased absorption intensity, as shown in Fig. 5c. As the incubation time increases from 40 to 120 min, the absorption peak intensity also increases. However, at 140 min, the absorbance or peak intensity decreased, likely due to nanoparticle aggregation, shrinkage, or changes during extended storage^40,42^. The optimal reaction time for the maximum yield of CuO NPs was found to be 120 min.

### Characterization of CuONPs

#### UV–Vis spectroscopy

**Fig. 6:**
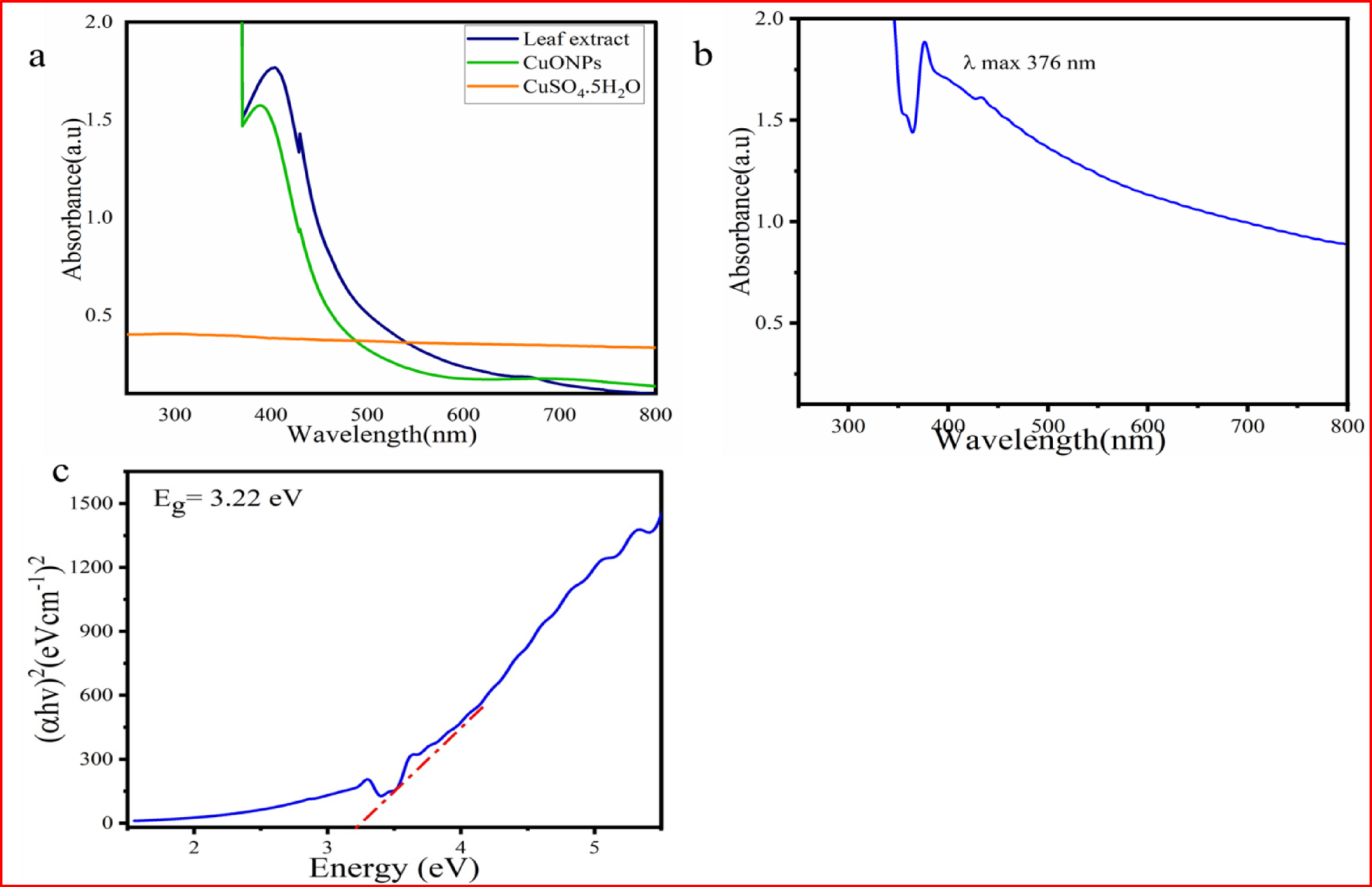
UV–Vis spectra of (**a**) CuSO_4_·5H_2_O precursor, leaf extract of *P. zeylanica*, CuO NPs before calcination, (**b**) CuO NPs after calcination, and (**c**), estimated band gaps using Taucs equation of the synthesized CuO NPs.

The optical absorption characteristics of the synthesized CuO NPs were subjected to scanning through a UV‒vis spectrophotometer in the wavelength range of 200–800 nm. The UV–Vis spectra of the freshly prepared CuONPs from *P. zeylanica* leaf extract and CuSO_4_·5H_2_O solution before calcination are presented in Fig. 6a. According to the figure, the synthesized CuONPs exhibited a maximum wavelength of 390 nm before calcination, which indicates the formation of CuO NPs. Additionally, the *P. zeylanica* leaf extract exhibited a significant absorbance at 402 nm. The peak of the plant extract shifted from 402 to 390 nm, indicating the formation of CuO NPs.

As shown in Fig. 6b, after calcination, the UV–vis absorption spectrum of the synthesized CuO NPs exhibited a characteristic band at 376 nm. This absorption peak is attributed to the interband transition of electron and surface electron fluctuation, where electrons are excited between energy levels upon exposure to electromagnetic radiation on the NP surface. Similarly, the UV–Vis spectrum of the CuO NPs biosynthesized using *Mussaenda frondosa* extracts showed absorption in the range of 377–382 nm^33^, while those synthesized using *Capsicum frutescens* exhibited an absorption peak at 379 nm^44^. Additionally, a surface plasmon resonance (SPR) peak at 373 nm was observed for CuO NPs synthesized using the leaf extract of *Nilgirianthus ciliates,* confirming the formation of CuONPs^45^.

The optical band gap was calculated from Tauc’s plot using Eq. (2)^7^.

2 $$\alpha h\nu = \kappa \left( {hv - Eg} \right)^{{\text{n}}}$$

The band gap energies were estimated by plotting (αhν)^2^ versus the photon energy (hν), as shown in Fig. 6c. The band gap energy of 3.22 eV was obtained for the CuO NPs, indicating quantum confinement effects resulting from variations in morphology, surface structure, and particle size^16^. The obtained band gap for CuO NPs deviated from the band gap of bulk CuO, which is 1.77 eV^7^. These values are in good agreement with the previous reports. The band gap of CuO NPs synthesized using *Tribulus terrestris* seed extract was 3.1 eV^21^. Similarly, the band gap energy of CuO NPs synthesized using *Clausena anisata* and E*uphorbia abyssinia* extract was reported as 3.25 eV and 3.11 eV, respectively^46^.

**Fig. 7:**
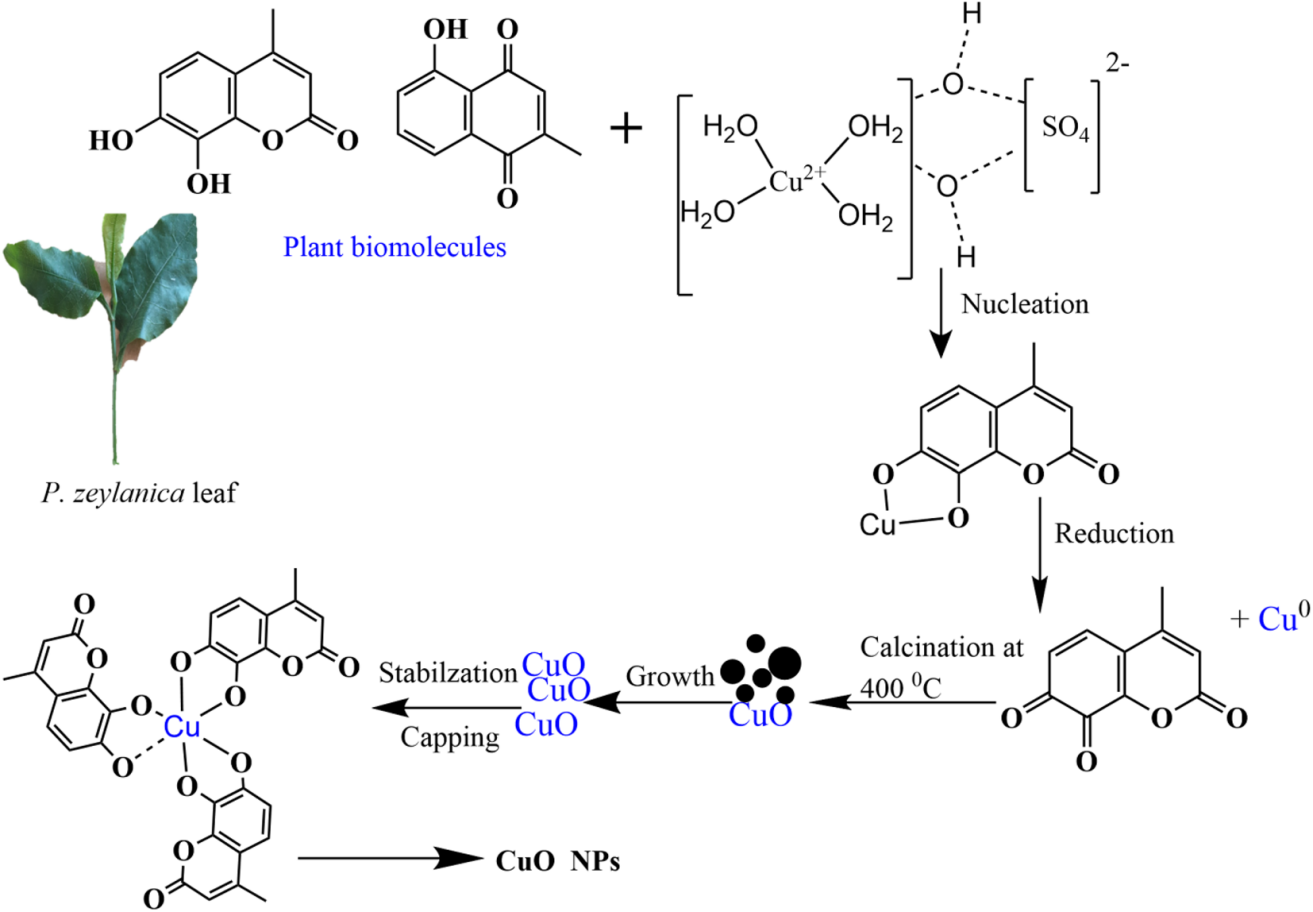
Proposed reaction mechanism of the formation of CuO NPs using leaf extract of *P. zeylanica.*

The mechanism of green synthesis of CuO NPs using plant extracts is complex due to the diverse range of phytoconstituents present. These compounds are believed to act as reducing, stabilizing, capping, or chelating agents during the formation of CuO NPs. When the leaf extract is added to the CuSO_4_·5H_2_O solution, Cu^2+^ ions interact with phytoconstituents, forming an intermediate Cu^2+^ phytochemical complex. The most plausible mechanism for the reduction of Cu^2+^ and the synthesis of CuO NPs in the presence of *P. zeylanica* aqueous leaf extract is depicted in Fig. 7.

#### Fourier transforms infrared spectroscopy

FT-IR spectra were used to identify functional groups in both the synthesized CuO NPs and *P. zeylanica* aqueous extract. The pellets of CuO NPs were prepared using the KBr disk method, with the wavenumber ranging from 400 to 4000 cm^−1^.

**Fig. 8:**
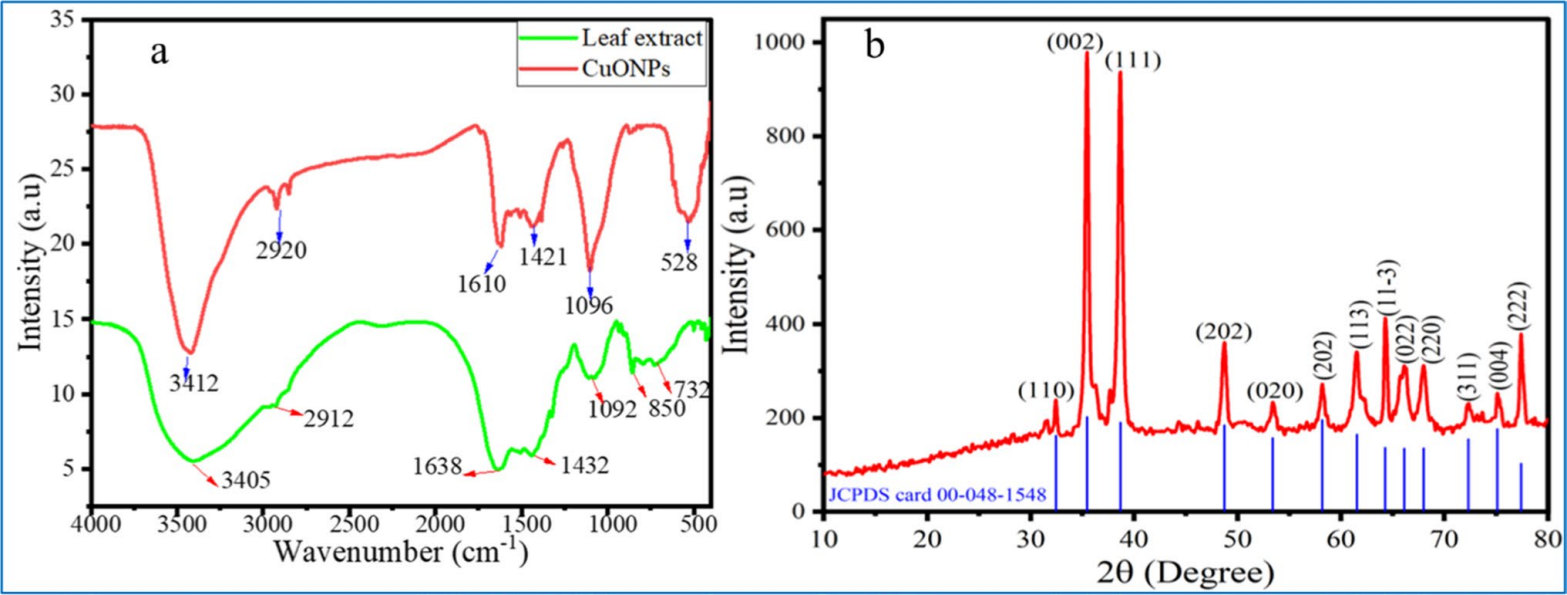
(**a**) FT-IR spectra of CuO NPs and (**b**) XRD of leaf extract *P. zeylanica* synthesized CuONPs.

In this study, the characteristic bands of the *P. zeylanica* aqueous extract were observed at 3405, 2912, 1638, 1432, 1092, 850, and 732 cm^−1^, while the CuO NPs exhibited peaks at 3412, 2920, 1610, 1421, 1096, and 528 cm^−1^, as shown in Fig. 8a. Based on the FTIR spectrum, the intense and broad peak in the 3412–3405 cm^−1^ range represents the O–H group characteristic of phenols, which was present in both the extract and CuO NPs spectra^17,47^. The band at 2920 -2912 cm^−1^ is associated with the stretching vibrations of CH, CH_2_, and CH_3_ groups, commonly found in carbohydrates, flavonoids, and polyphenols^48^. The peaks observed between 1638 and 1421 cm^−1^ are attributed to the C=O/C=C vibration of aromatic polyphenols with phenyl rings^15,47^.

Additional peaks at 1096–1092 and 850 cm^−1^ were attributed to C − O and C − H bending vibrations, respectively^16^. The peak at 732 cm^−1^ corresponds to aromatic ring C–H bending^19^. Furthermore, the peak at 528 cm^−1^ suggests the stretching vibration of Cu–O bonds, supporting the monoclinic phase of CuONPs. These values align with previous literature^16,33,47,49^.

Minor shifts were observed in the frequencies from the plant extract (3405, 2912, 1638, 1432, 1092, and 850 cm^−1^) to the CuO NPs (3412, 2902, 1610, 1421, 1096, and 528 cm^−1^), indicating that the functional groups in the plant extract acted as reducing agents during the synthesis of CuO NPs, causing alterations in the FTIR spectrum. This suggests that phytochemicals played a key role in capping and stabilizing agents CuO NPs during their green synthesis^17^.

#### X-ray diffraction analysis

In this study, the major diffraction peaks observed in the XRD diffractogram are shown in Fig. 8b at 32.42°, 35.44°, 38.66°, 48.72°, 53.40°, 58.18°, 61.52°, 64.28°, 66.10°, 67.98°, 72.30°, 75.10°, and 77.40° correspond to crystallographic planes (110), (002), (111), (202), (020), (202), (113), (11–3), (022), (220), (311), (004), and (222), respectively, which reveals the monoclinic structure of CuO with space group C2/c (JCPDS card no. 00–048-1548, Tenorite‐C_2_/c). These patterns are in good agreement with the earlier studies on the green synthesis of CuO NPs^20,50,51^.

The average crystallite size of the CuO NPs was estimated using Debye Scherer’s Eq. (3)^52^, as shown below.

3 $${\text{D}} = \frac{{{\text{k}}\lambda }}{\beta \cos \theta }$$

where D is the particle size (nm), λ is the wavelength of the X-ray (λ = 1.54056 ˚A), ĸ is the Scherrer constant (0.94), β is the full-width at half maximum (FWHM), and θ is the diffraction angle (half of the Bragg angle) that corresponds to the lattice plane. Additionally, the dislocation density (δ) of biosynthesized CuONPs was determined using Eq. (4)^7^.

4 $$\partial = \frac{1}{{D^{2} }}$$

where D is the average particle size.

The calculated average crystal size (D) of the synthesized CuO NPs was approximately 25.15 nm, as shown in Table 2. Comparable average crystal sizes of CuO NPs, ranging from 20 to 30 nm, have been exclusively reported by^5,20,53^, and^3^ in CuO NPs synthesized using *Abelmoschus esculentus, Bacopa monnieri, Celastrus paniculatus,* and *Morinda citrifolia* leaf extract, respectively. The percent crystallinity of the biosynthesized CuO NPs was also calculated through the following Eq. (5) 54.

**Table 2:** XRD data and calculation of the average size of synthesized CuO NPs.

*hkl*	2*θ* (degree)	D-spacing(nm)	FWHM, *β* (deg)	D (nm)	Dislocation density (*δ*)
(110)	32.42	0.276	0.275	31.38	0.0010
(002)	35.44	0.253	0.367	23.71	0.0018
(111)	38.66	0.233	0.445	19.75	0.0026
(202)	48.72	0.187	0.453	20.10	0.0025
(020)	53.4	0.171	0.355	26.16	0.0015
(202)	58.18	0.158	0.401	23.66	0.0018
(113)	61.52	0.151	0.502	19.24	0.0027
(11-3)	64.28	0.145	0.275	35.70	0.0008
(022)	66.1	0.141	0.600	16.50	0.0037
(220)	67.98	0.138	0.505	19.82	0.0025
(311)	72.3	0.131	0.355	28.95	0.0012
(004)	75.1	0.126	0.480	21.80	0.0021
(222)	77.4	0.123	0.265	40.15	0.0006
Average crystal size					25.15 nm

5 $$\text{Percent Crystallinity}=\frac{\text{Area of Crystalline peak}}{\text{Area of all peaks }(\text{Crystalline}+\text{Amorphous})}\text{ x }100$$

The crystallinity of the CuO NPs was determined to be 93.78%, indicating a low amorphous content. The high crystallinity and low dislocation density further confirm the excellent structural quality of the synthesized CuO NPs. Additionally, the prominent diffraction intensities observed at 2θ values of 35.51° and 38.82° indicate the high crystallinity of the synthesized CuO NPs^55^.

#### Thermogravimetric analysis

The existing biomolecules, thermal stability, and energy changes (gains or losses) during the synthesis process of the biosynthesized CuO NPs were studied and examined using TGA/DTA under an N_2_ atmosphere (DTG-60H, Shimadzu Co., Japan), with the temperature range set from 0 to 800 °C at the rate of 15 °C/min.

**Fig. 9:**
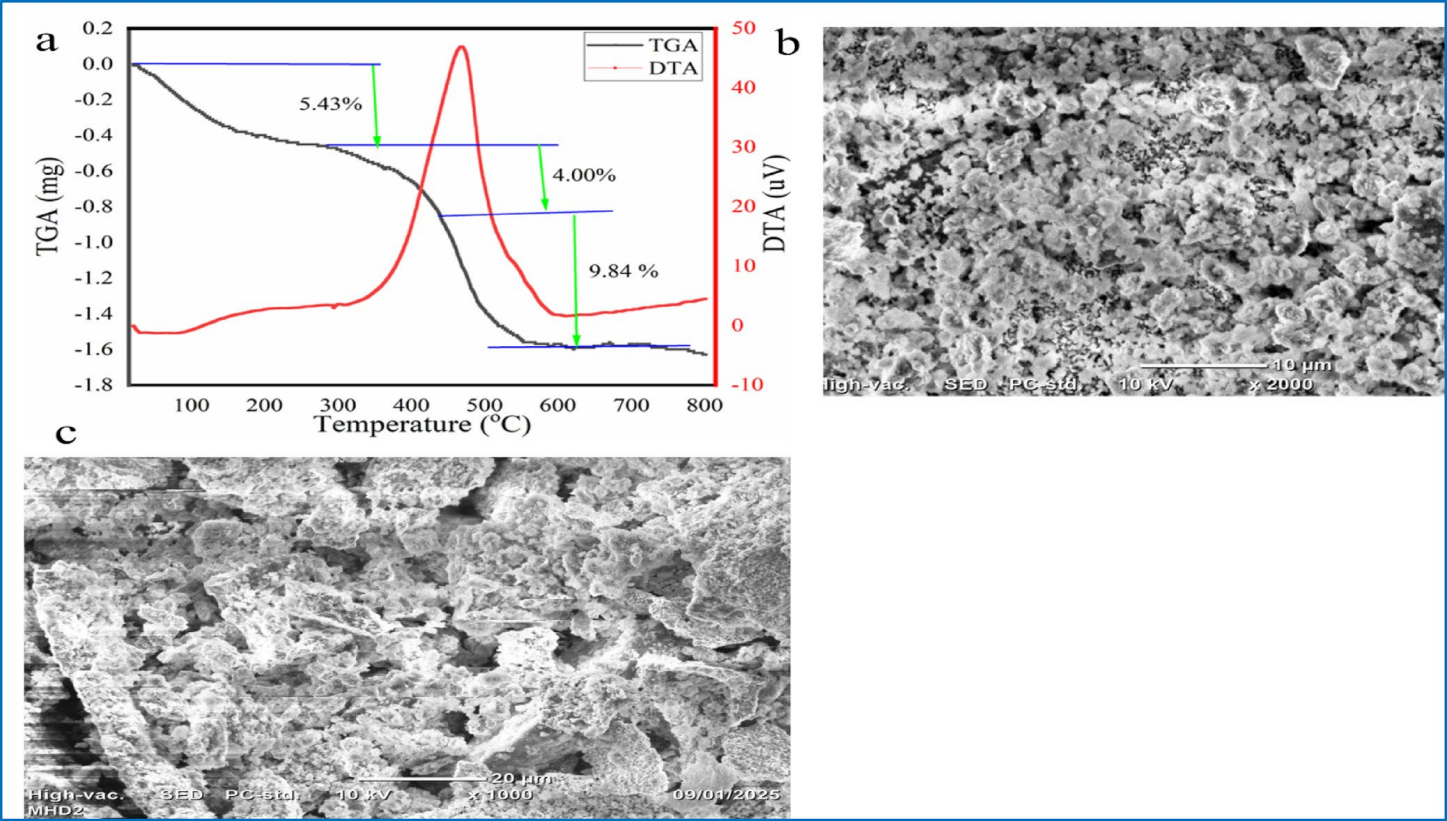
(**a**) TGA and DTA curves, (**b**) SEM images with × 2000 magnification, and (**c**) SEM images with × 1000 magnification of CuO NPs synthesized using *P. zeylanica* leaf extract.

As shown in Fig. 9a, the TGA curve revealed a three-phase decomposition of CuO NPs. The initial 5.43% reduction in weight occurred between 21 °C and 232 °C due to the loss of volatile chemical components and evaporation moisture content. Following the initial weight loss, the second and third weight losses were seen to be 4.00% and 9.84% from 232 to 435 °C and 435 to 600 °C, respectively, due to the combustion of the biomolecules and complete disintegration of bioorganic constituents that were capped on the surface of the CuO NPs. After 600 °C, no more weight loss was recorded when heated to 800 °C^17,56^. The total weight loss was 19.3%, corresponding to 80.7% CuO content, indicating high thermal stability. The DTA curve shows two mass losses (21–435 °C) with an endothermic peak, followed by an exothermic peak (435–600 °C), indicating the disintegration of bioorganic phytoconstituents in CuO NPs.

Comparatively, CuO NPs synthesized using *Bacopa monnieri* and *Artemisia abyssinica* showed weight losses of 33.3%^5^ and 21.64%^38^, respectively.

#### Scanning electron microscopy

The morphology and surface features of CuO NPs were examined using SEM, as shown in Fig. 9b. SEM analysis revealed that the CuO NPs exhibited predominantly spherical, sheet-like, and irregular shapes, with some degree of agglomeration forming crystal clusters. The observed agglomeration suggests increased surface reactivity, potentially due to high-temperature calcination^57^.

Furthermore, the particles appear evenly dispersed across the surface with minimal agglomeration, which may be attributed to the presence of stabilizing agents. The presence of active groups in the *P. zeylanica* aqueous plant extract may also contribute to agglomeration, as the phytochemicals involved in bioreduction and capping play a role in the reaction environment.

### Quantitative analysis of phytochemicals screening

In this study, the TPC and TFC of *P. zeylanica* leaf aqueous extracts and CuO NPs were quantified, as shown in Table 3. The TPC value of CuO NPs was found to be 8.48 ± 0.07 mg GAE/g extract, while the aqueous extract exhibited a significantly higher value of 35.27 ± 0.26 mg GAE/g extract. The higher TPC value in the aqueous extract may be attributed to enhanced phenolic biosynthesis in *P. zeylanica.*

**Table 3:** Quantitative phytochemical analysis of *P.zeylanica* leaf extracts and CuONPs.

Sample	TPC (mg GAE/g dry extract)	TFC (mg QE/g dry Extract)
Plant extracts	35.27 ± 0.26	26.76 ± 0.14
CuO NPs	8.48 ± 0.07	6.84 + 0.37

The aqueous extract also had the highest TFC (26.76 ± 0.14 mg QE/g extract), whereas the CuO NPs exhibited a lower TFC (6.84 ± 0.37 mg QE/g extract). This difference could be because flavonoids and phenolics often contain hydroxyl groups, which can be oxidized or degraded under high temperatures or oxidative environments, thereby reducing their overall content in the final CuO NP product. A similar study on the synthesis of CuO NPs using *Bergenia ciliate* aqueous extracts reported TPC and TFC values of 49.2 ± 1.7 mg GAE/g and 22.1 ± 1.0 mg QE/g extract, respectively^58^.

### Determination of antioxidant activity

#### DPPH radical scavenging assays

**Fig. 10:**
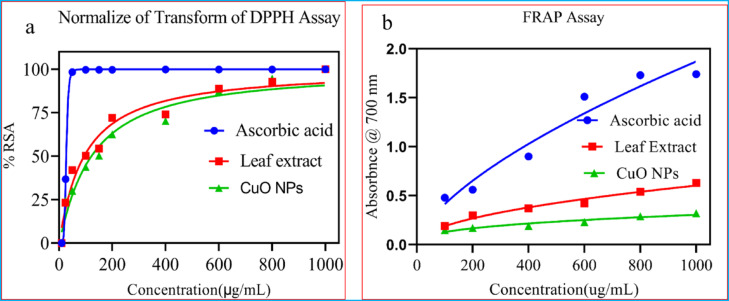
Antioxidant profiles of (**a**) DPPH and (**b**) reducing power ability of ascorbic acid, leaf extract, and synthesized CuONPs.

The antioxidant potential of synthesized CuO NPs and *P. zeylanica* leaf extract was assessed using DPPH radical scavenging activity, as illustrated in Fig. 10a. The IC_50_ value of standard ascorbic acid was determined to be 27.08 ± 0.15 μg/mL. The aqueous leaf extract of *P. zeylanica* exhibited an IC_50_ of 97.28 ± 1.85 μg/mL, indicating higher antioxidant activity compared to the green synthesized CuO NPs, which showed an IC_50_ of 123.77 ± 1.96 μg/mL. A lower IC_50_ value indicates stronger DPPH radical scavenging ability, while a higher IC_50_ value indicates reduced scavenging potential^33^. In this study, the antioxidant activity followed the order of ascorbic acid > leaf extract > CuO NPs.

The antioxidant activity of the CuO NPs synthesized using *P. zeylanica* was compared with that of CuO NPs synthesized from other plant extracts, as shown in.

Table 4. The IC_50_ value of the CuO NPs in the current study was higher than that reported by^44^, 34.97 μg/mL, but lower than that reported by^59^, 131.54 μg/mL. These findings suggest that, in comparison to other studies, the synthesized CuO NPs exhibited notable DPPH radical scavenging activity.

**Table 4:** Comparison of antioxidant activity of *P. zeylanica*-synthesized CuO NPs to other green synthesized CuONPs.

Plant extract	Material	IC_50_ (μg/mL)	References
*Plumbago zeylanica*	CuO NPs	123.77 ± 1.96	Current study
*Bergenia ciliate*	CuO NPs	91.2 μg	^58^
*Mussaenda frondosa*	CuO NPs	1536–1570	^33^
*Ligustrum lucidum*	CuO NPs	63.45	^57^
*Capsicum frutescens*	CuO NPs	40	^32^
*Suaeda maritime*	CuO NPs	28.05	^60^
*Artemisia abyssinica*	CuO NPs	5.75	^38^
*Tribulus terrestris*	CuO NPs	51.53	^55^

#### Ferric reducing antioxidant power assay

During the FRAP assay, the presence of electron-donating compounds facilitated the conversion of the Fe^3+^ ferricyanide complex to Fe^2+^ ferrous ions^61^. As illustrated in Fig. 10b**,** the ferric-reducing power activity of both the extracts and synthesized CuO NPs was evaluated at concentrations ranging from 100 to 1000 μg/mL, demonstrating a general increase in activity with higher concentrations. Both the synthesized CuO NPs and the leaf extract in this study exhibited dose-dependent radical scavenging properties.

The ascorbic acid had better free radical-reducing power activity, followed by *P. zeylanica* leaf extract and then the CuO NPs with increasing concentrations. An increased absorbance of the reaction mixture corresponded to enhanced reducing power^61^. This pattern suggests that the leaf extract exhibits more activity than the CuO NPs, supporting its potential as an efficient antioxidant, whereas ascorbic acid, as a positive control, has superior reducing power. The FRAP assay aligns with the previous finding of the concentration-dependent activity pattern^62^.

### Antibacterial activity

**Fig. 11:**
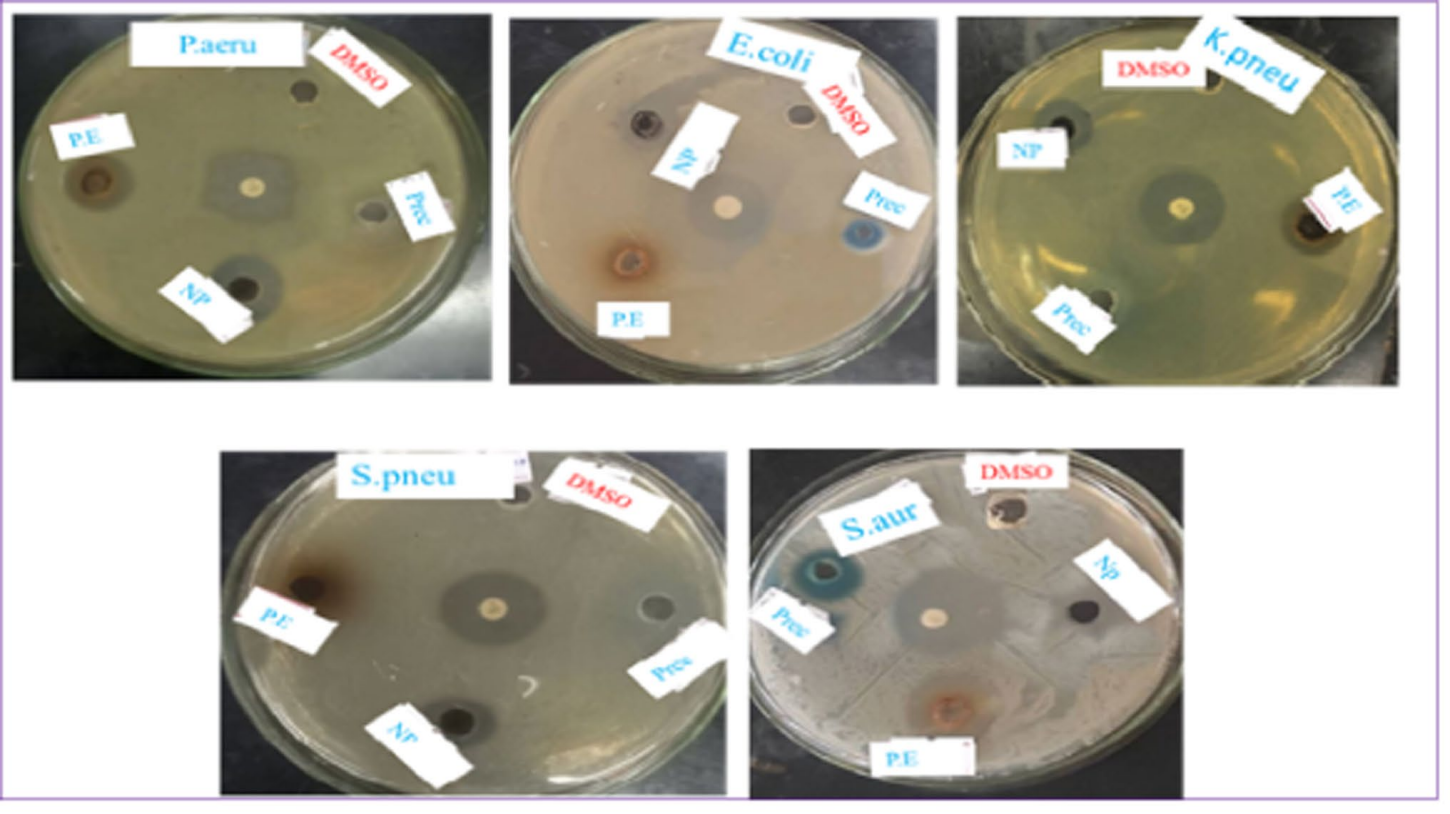
Zone of inhibition of synthesized CuO NPs (NP), *P .zeylanica* extracts (P.E), and CuSO_4_·5H_2_O (Prec) against various pathogenic bacterial strains.

The antibacterial properties of CuO NPs, *P. zeylanica* leaf extract, and CuSO_4_·5H_2_O were tested against Gram-positive (*S. aureus*, *S. pneumonia*) and Gram-negative (*P. aeruginosa*, *E. coli*, *K. pneumonia*) bacteria using the agar well diffusion method. DMSO served as a negative control, and ciprofloxacin was used as a positive control, with each experiment conducted in triplicate to ensure reliable results. CuO NPs exhibited the highest antibacterial activity against *P. aeruginosa* (20.30 ± 0.57 mm), followed by *E. coli* (19.33 ± 0.57 mm) and *K. pneumonia* (16.5 ± 0.50 mm), showing a synergistic effect compared to the *P. zeylanica* leaf extract and CuSO_4_·5H_2_O. The smallest inhibition zone (14.50 ± 0.57 mm) was observed for *S. aureus*. The antibacterial effectiveness of CuONPs followed the order of *P. aeruginosa* > *E. coli* > *K. pneumonia* > *S. pneumonia* > *S. aureus*. The leaf extract showed the highest inhibition against *P. aeruginosa* (15.33 ± 0.577 mm), *E. coli* (14.33 ± 0.577 mm), and *K. pneumonia* (12.16 ± 0.28 mm), while CuSO_4_·5H_2_O showed lower activity across all bacterial strains. The smallest inhibition zone for CuSO_4_·5H_2_O was against *S. aureus* (7.16 ± 0.57 mm) as shown in Table 5 and Fig. 11. The mechanism underlying the antibacterial activity of CuO NPs is influenced by their particle size, structural characteristics, and concentration^3^. CuO NPs exhibit enhanced sensitivity and antimicrobial activity relative to their bulk forms. Their strong antibacterial effects are largely due to their nanoscale size and large surface area, which promote effective interaction with bacterial cell membranes, resulting in cellular disruption and death^58^. Furthermore, the pronounced activity against both Gram-positive and Gram-negative bacteria may be due to the generation of reactive oxygen species (ROS) by CuO^17^. ROS, such as OH− radicals, superoxide ions (O_2_^2−^), and hydrogen peroxide (H_2_O_2_), interact with the bacterial cell membrane. These ROS disrupt the cell wall and penetrate the cell interior. Additionally, electrostatic interactions between the bacterial surface and the nanoparticles contribute to cell wall rupture^32^.

**Table 5:** Evaluation of the antibacterial effect of the *P .zeylanica* extracts, CuO NPs, and copper sulfate against five pathogenic bacteria.

	Inhibition zone (mm) against bacterial strains				
	*K. pneumonia*	*S. pneumonia*	*S. aureus*	*E.coli*	*P. aeruginosa*
Leaf extract	12.16 ± 0.28	10.67 ± 0.57	10.33 ± 0.57	14.33 ± 0.57	15.33 ± 0.57
CuO NPs	16.50 ± 0.50	15.30 ± 0.57	14.50 ± 0.50	19.33 ± 0.57	20.30 ± 0.57
CuSO_4_·5H_2_O	8.33 ± 0.57	7.33 ± 0.57	7.16 ± 0.28	11.33 ± 0.57	11.67 ± 0.57
Ciprofloxacin	20.67 ± 0.57	19.67 ± 0.57	22.33 ± 0.57	22.67 ± 0.577	24.33 ± 0.57
DMSO	0.00	0.00	0.00	0.00	0.00
*P* value	< 0.05	< 0.05	< 0.05	< 0.05	< 0.05

Table 5 and Fig. 11. A significant difference (*p* < 0.05) was observed between the antibacterial activities of CuO NPs, extract, and CuSO_4_·5H_2_O. The results suggest that gram-negative bacteria are more susceptible to CuO NPs than gram-positive bacteria, likely due to their thinner peptidoglycan layer, which facilitates easier NP penetration^63^. In contrast, the increased resistance observed in Gram-positive bacteria can be attributed to their thicker peptidoglycan layer, which acts as a protective barrier against NPs infiltration^64^. The synthesized CuONPs in this study showed strong antibacterial activity compared to previous research, as shown in Table 6. The mechanism underlying the antibacterial activity of CuO NPs is influenced by their particle size, structural characteristics, and concentration^3^. CuO NPs exhibit enhanced sensitivity and antimicrobial activity relative to their bulk forms. Their strong antibacterial effects are largely due to their nanoscale size and large surface area, which promote effective interaction with bacterial cell membranes, resulting in cellular disruption and death^58^. Furthermore, the pronounced activity against both Gram-positive and Gram-negative bacteria may be due to the generation of reactive oxygen species (ROS) by CuO^17^. ROS, such as OH^−^ radicals, superoxide ions (O_2_^2−^), and hydrogen peroxide (H_2_O_2_), interact with the bacterial cell membrane. These ROS disrupt the cell wall and penetrate the cell interior. Additionally, electrostatic interactions between the bacterial surface and the nanoparticles contribute to cell wall rupture^32^.

**Table 6:** Comparison of antibacterial activity of green synthesized CuO NPs.

Plant extract	Pathogen name	Zone inhibition (mm)	References
*P.zeylanica*	*P. aeruginosa*	20.30	Present study
*Aerva javanica*	*S. aureus*	9.00	^18^
*Thespesia populnea*	*P. aeruginosa*	8.50	^65^
*Bergenia ciliate*	*S. aureus*	17.80	^58^
*Vernonia amygdalina*	*E. aerogene*	15.00	^51^
*Balanites aegyptiaca*	*B. substilis*	13.00	^2^
*Aloe vera*	*Listeria monocytogene*	15.00	^16^
*Morinda citrifolia*	*Bacillus subtilis*	13.60	^3^
*Acer palmatum*	*E. coli*	23.00	^15^

## Conclusions

The present study reports a cost-effective and eco-friendly green synthesis of CuO NPs using aqueous extracts of *P. zeylanica*. Phytochemical analysis of the leaf extract revealed tannins, phenols, saponins, flavonoids, terpenoids, steroids, coumarins, and cardiac glycosides as the major bioactive compounds responsible for nanoparticle formation, acting as natural reducing and capping agents. The synthesis was optimized by varying parameters including CuSO_4_·5H_2_O concentration, extract-to-precursor volume ratio, temperature, pH, and reaction time, resulting in stable CuO NPs with enhanced yield and quality, evidenced by a distinct color change. The synthesized CuONPs were characterized by UV–Vis, FTIR, SEM, XRD, and TGA/DTA. UV–Vis analysis showed an absorbance peak at 376 nm, confirming CuO NPs formation. XRD patterns indicated a monoclinic structure with a crystal size of 25.15 nm and crystallite purity of 93.78%. FTIR analysis identified a hydroxyl group (3412–3405 cm⁻^1^), indicating Cu ion reduction and nanoparticle stabilization. SEM investigates the morphology of CuO NPs. The antioxidant activity of CuO NPs showed lower radical scavenging potential than ascorbic acid, but they can still combat oxidative stress. CuO NPs demonstrated strong antibacterial activity against *S. aureus*, *S. pneumonia*, *E. coli*, *P. aeruginosa*, and *K. pneumonia*, highlighting their potential for treating bacterial infections. This study suggests that green-synthesized CuO NPs are effective as both antioxidants and antibacterial agents.

## Data Availability

The data produced in this study are available within the manuscript.
